# Failure of Isoniazid Chemoprophylaxis during Infliximab Therapy

**DOI:** 10.3201/eid1309.070070

**Published:** 2007-09

**Authors:** Manuel L. Fernández-Guerrero, Jaime Esteban, Carlos Acebes, Miguel Górgolas

**Affiliations:** * University of Madrid, Madrid, Spain

**Keywords:** *Mycobacterium kansasii*, Infliximab therapy, TNF-blockers, tuberculosis, letter

**To the Editor:** A patient with ankylosing spondylitis was treated with infliximab, a tumor necrosis factor (TNF) blocker that has been associated with reactivation of latent tuberculosis (TB). Because of reactivity in testing with purified protein derivative, isoniazid chemoprophylaxis was started 2 weeks before infliximab therapy. Four months later, a cavitary lung infection developed in the patient, caused by isoniazid-resistant *Mycobacterium kansasii.*

To our knowledge, this is the first documented case of failure of isoniazid prophylaxis due to the emergence of isoniazid-resistant mycobacteria in patients receiving infliximab therapy. TNF blockers have contributed to the control of rheumatic diseases (*1*). Many of the damaging inflammatory mechanisms that they inhibit are important in maintaining TB in the latent phase. Consequently, drugs that target TNF functions have been associated with an increased risk of TB (*2*). For these reasons, prophylactic chemotherapy should be offered to patients with latent TB (*3*). We show the failure of isoniazid chemoprophylaxis in a patient receiving infliximab therapy in whom lung infection developed, caused by isoniazid-resistant *M. kansasii.*

A 39-year-old man with ankylosing spondylitis was admitted to Jimenez Diaz Foundation hospital, Madrid, because of fever and lung infiltrates. He had been receiving anti-inflammatory drug therapy without amelioration of his symptoms. Therefore, treatment with infliximab was considered. Fifteen years before, the patient’s father had had pulmonary TB caused by *M. tuberculosi*s that was susceptible to first-line antituberculous drugs, and the patient was given chemoprophylaxis with isoniazid, 300 mg/day, during a 9-month period. Before beginning infliximab therapy, the patient was again given chemoprophylaxis with isoniazid, 300 mg/day, because a tuberculin test with 5 units of purified protein derivative showed an induration of 18 mm at 72 hours. Results of chest radiographs were normal, and cultures for mycobacteria were negative. Results of HIV testing were also negative.

After 4 months of infliximab therapy, fever, cough, and sputum production developed. New radiographs showed bilateral upper lung field infiltrates with cavitary lesions. Three acid-fast stains of sputum were positive, and treatment with rifampin, isoniazid, pyrazinamide, and ethambutol was started.

A heavy growth of photochromogenic mycobacteria was detected in 3 sputum cultures. The isolate was identified as *M. kansasii* genotype 1 by using common biochemical tests and PCR–restriction fragment length polymorphism analysis of the *hsp*65 gene (*4*). Susceptibility tests showed resistance to isoniazid (>5 µg/mL), streptomycin, pyrazinamide, p-amino-salicylic acid, and kanamycin but susceptibility to rifampin, ethambutol, and fluoroquinolones.

Treatment was continued with a combination of rifampin, levofloxacin, and ethambutol. Sputum cultures taken after 4, 6, and 9 months of antimicrobial drug therapy were negative. After 20 months of treatment, the patient was doing well with a partial resolution of lung infiltrates, and new cultures were negative.

Isoniazid chemoprophylaxis can effectively lessen the likelihood of active TB in patients treated with TNF antagonists (*5*). However, at least 1 failure of TB chemoprophylaxis in a severely immunocompromised patient treated with infliximab and methotrexate has been published (*6*). Our patient is unique because the mycobacterial lung infection seemed to emerge as a result of the lack of activity of isoniazid chemoprophylaxis due to resistance of the infecting organism.

Decreased susceptibility to isoniazid among *M. kansasii* isolates is common (*7**,**8*), and this microorganism is naturally resistant to pyrazinamide (*9*). This pattern of resistance is a serious obstacle for the use of these drugs in monotherapy or when combined with rifampin in the prevention of lung disease caused by *M. kansasii* (*10*)*.*

The source of the infection in this patient is unknown. In a large series of infectious diseases associated with infliximab therapy, nontuberculous mycobacteria were isolated in 9% of the patients who had mycobacterial diseases (*2*). As in our patient, these infections developed shortly after initiation of treatment with infliximab, which suggests that reactivation of a latent infection is the most probable origin of the disease. Although a mildly positive tuberculin skin test result can be observed in patients infected with atypical mycobacteria, the strong reaction seen in this patient suggests a latent infection with *M. tuberculosis* (*10*). We could speculate on the possibility of a double infection with *M. tuberculosis* (contracted through household contacts with his father) and *M. kansasii* through environmental exposure. In this scenario, isoniazid chemoprophylaxis could have prevented the former but not the latter.

In summary, failure of isoniazid chemoprophylaxis can be anticipated in patients who initiate treatment with infliximab and who have latent infections due to *M. kansasii*. Despite routine antituberculous chemoprophylaxis, patients receiving infliximab therapy should be carefully evaluated for lung infection caused by atypical mycobacteria.
